# Supplementary material for: Real-Life Effectiveness of Subcutaneous Immune Therapy with Carbamylated Monomeric Allergoids on Mite, Grass, and Pellitory Respiratory Allergy: A Retrospective Study
Source: J Clin Med. 2022 Dec 12;11(24):7384. doi: 10.3390/jcm11247384 (PMC9781390; doi:10.3390/jcm11247384)
Supplement: Supplementary file 1 [file jcm-11-07384-s001.zip › supplementary files/Supplementary file 3_asthma questionnaire.pdf]

DEAR PATIENT, PLEASE FILL IN THE FOLLOWING QUESTIONNAIRE

Write the initials of your first name and surname:

First letter of first name | First letter of surname

Age:

Sex:

- ◯ Man
- ◯ Woman
- ◯ Other

Do you remember what disease you were prescribed specific subcutaneous immunotherapy for?

- ☐ Rhinitis (cold) and/or conjunctivitis
- ☐ Asthma
- ☐ Both

Do you remember if you had prick tests?

- ☐ I don't remember
- ☐ Yes, and I only tested positive for the allergen for which I received immunotherapy
- ☐ Yes, and I tested positive also for other allergens
- ☐ No

Do you remember what allergen you were prescribed specific subcutaneous immunotherapy for?

- ☐ Dust mite
- ☐ Grass
- ☐ Pellitory

What therapeutic regimen did you follow?

- ☐ Periodic injections all year round
- ☐ Injections before and during the pollen season

Was it the first time you received specific subcutaneous immunotherapy?

- ☐ Yes
- ☐ No, the second time (for the same allergen)
- ☐ No, the third time (for the same allergen)
- ☐ No, but previously for a different allergen

CONTINUED

WE SHALL NOW ASK YOU SOME QUESTIONS ABOUT THE PERIOD LEADING UP TO THE START OF THE TREATMENT WITH SPECIFIC SUBCUTANEOUS IMMUNOTHERAPY

ASTHMA

What was the severity of symptom you suffered from the **year before** the start of treatment? Mark a point on the line below, bearing in mind that the left end indicates that you had no symptoms and the right end indicates that you suffered serious symptoms.

5.00

I have no symptoms — **extremely troublesome or serious symptoms**

During the year prior to starting treatment, did you have periods of daytime symptoms for at least two months?

- ☐ Yes
- ☐ No

During the **year before** the start of treatment, did your symptoms wake you up frequently during the night?

- ☐ Yes
- ☐ No

During the **year before** the start of treatment, did you use bronchodilators (e.g. salbutamol) as needed several times for at least two month periods?

- ☐ Yes
- ☐ No

During the **year before** the start of treatment, did your asthma interfere frequently with your normal activities?

- ☐ Yes
- ☐ No

During the **year before** the start of treatment, how many asthma exacerbations requiring systemic corticosteriods (from os or by injection) did you suffer?

☐ 0 ☐ 1 ☐ 2 ☐ 3 ☐ 4 ☐ 5 ☐ 6 ☐ 7 ☐ 8

CONTINUED

WE SHALL NOW ASK YOU SOME QUESTIONS ABOUT THE FIRST YEAR OF TREATMENT WITH SPECIFIC SUBCUTANEOUS IMMUNOTHERAPY

ASTHMA

What was the severity of symptom you suffered from the **first year** of treatment? Mark a point on the line below, bearing in mind that the left end indicates that you had no symptoms and the right end indicates that you suffered serious symptoms.

5.00

I have no symptoms **extremely troublesome or serious symptoms**

During the **first year** of treatment, did you get daytime symptoms for a two months period?

☐ Yes

☐ No

During the **first year** of treatment did the symptoms wake you up frequently during the night?

☐ Yes

☐ No

During the **first year** of treatment, did you use bronchodilators (e.g. salbutamol) as needed several times for at least two month periods?

☐ Yes

☐ No

During the **first year** of treatment, did your asthma interfere frequently with your normal activities?

☐ Yes

☐ No

During the **first year** of treatment, how many asthma exacerbations requiring systemic corticosteriods (from os or by injection) did you suffer?

☐ 0 ☐ 1 ☐ 2 ☐ 3 ☐ 4 ☐ 5 ☐ 6 ☐ 7 ☐ 8

CONTINUED

WE SHALL NOW ASK YOU SOME QUESTIONS ABOUT THE END OF TREATMENT WITH SPECIFIC SUBCUTANEOUS IMMUNOTHERAPY

ASTHMA

What was the severity of symptom you suffered at the **end** of the treatment? Mark a point on the line below, bearing in mind that the left end indicates that you had no symptoms and the right end indicates that you suffered serious symptoms.

5.00

I have no symptoms — **extremely troublesome or serious symptoms**

At the **end** of treatment, did you get daytime symptoms for a two months period?

☐ Yes

☐ No

At the **end** of treatment did the symptoms wake you up frequently during the night?

☐ Yes

☐ No

At the **end** of treatment, did you use bronchodilators (e.g. salbutamol) as needed several times for at least two month periods?

☐ Yes

☐ No

At the **end** of treatment, did your asthma interfere frequently with your normal activities?

☐ Yes

☐ No

At the **end** of treatment, how many asthma exacerbations requiring systemic corticosteriods (from os or by injection) did you suffer?

☐ 0 ☐ 1 ☐ 2 ☐ 3 ☐ 4 ☐ 5 ☐ 6 ☐ 7 ☐ 8

CONTINUED

WE SHALL NOW ASK YOU QUESTIONS ABOUT THE PERIOD OF DISCONTINUATION OF THE TREATMENT

How many years ago did you discontinue treatment?

☐ 1 year

☐ 2 years

ASTHMA

What was the severity of symptom **since you discontinued** the treatment? Mark a point on the line below, bearing in mind that the left end indicates that you had no symptoms and the right end indicates that you suffered serious symptoms.

5.00

I have no symptoms — **extremely troublesome or serious symptoms**

Since you discontinued treatment, have you had daytime symptoms for a two months period?

☐ Yes

☐ No

Since you discontinued treatment have the symptoms woken you up frequently during the night?

☐ Yes

☐ No

Since you discontinued treatment, did you use bronchodilators (e.g. salbutamol) as needed several times for at least two month periods??

☐ Yes

☐ No

Since you discontinued treatment, has your asthma interfered frequently with your normal activities?

☐ Yes

☐ No

Since you discontinued treatment, how many asthma exacerbations requiring systemic corticosteriods (from os or by injection) did you suffer?

☐ 0 ☐ 1 ☐ 2 ☐ 3 ☐ 4 ☐ 5 ☐ 6 ☐ 7 ☐ 8

CONCLUSIVE QUESTIONS

Are you satisfied with the treatment?

☐ Very unsatisfied

☐ Unsatisfied

☐ Satisfied

☐ Very satisfied

Indicate with a score from 1 to 5 you badly you have been disturbed during the past 2 weeks by:

	Not at all	A little	Quite a lot	A lot	A great deal
Blocking, dripping or itchy nose	1	2	3	4	5
Itching, lacrimation, burning or redness of the eyes	1	2	3	4	5
Difficulty concentrating	1	2	3	4	5
Wheezing, coughing, chest tightness or difficulty breathing	1	2	3	4	5
Disturbed sleep (e.g. night waking)	1	2	3	4	5
Having to avoid certain environments	1	2	3	4	5
Having to take medicinal products	1	2	3	4	5
Limitations to normal daytime activities (work, study, sport)	1	2	3	4	5

Enter your check code to complete the questionnaire

COMPLETE QUESTIONNAIRE
