# Supplementary material for: Real-Life Effectiveness of Subcutaneous Immune Therapy with Carbamylated Monomeric Allergoids on Mite, Grass, and Pellitory Respiratory Allergy: A Retrospective Study
Source: J Clin Med. 2022 Dec 12;11(24):7384. doi: 10.3390/jcm11247384 (PMC9781390; doi:10.3390/jcm11247384)
Supplement: Supplementary file 1 [file jcm-11-07384-s001.zip › supplementary files/Supplementary file 2_rhinocongiunctivitis questionnaire.pdf]

DEAR PATIENT, PLEASE FILL IN THE FOLLOWING QUESTIONNAIRE

Write the initials of your first name and surname:

First letter of first name | First letter of surname

Age:

Sex:

- ◯ Man
- ◯ Woman
- ◯ Other

Do you remember what disease you were prescribed specific subcutaneous immunotherapy for?

- ☐ Rhinitis (cold) and/or conjunctivitis
- ☐ Asthma
- ☐ Both

Do you remember if you had prick tests?

- ☐ I don't remember
- ☐ Yes, and I only tested positive for the allergen for which I received immunotherapy
- ☐ Yes, and I tested positive also for other allergens
- ☐ No

What allergen you were prescribed specific subcutaneous immunotherapy for?

- ☐ Dust mite
- ☐ Grass
- ☐ Pellitory

What therapeutic regimen did you follow?

- ☐ Periodic injections all year round
- ☐ Injections before and during the pollen season

Was it the first time you received specific subcutaneous immunotherapy?

- ☐ Yes
- ☐ No, the second time (for the same allergen)
- ☐ No, the third time (for the same allergen)
- ☐ No, but previously for a different allergen

CONTINUED

WE SHALL NOW ASK YOU SOME QUESTIONS ABOUT THE PERIOD LEADING UP TO THE START OF THE TREATMENT WITH SPECIFIC SUBCUTANEOUS IMMUNOTHERAPY

RHINOCONJUNCTIVITIS

What was the severity of nose and eye symptoms during the **year before** the start of treatment? Mark a point on the line below, bearing in mind that the left end indicates that you had no symptoms and the right end indicates that you suffered extremely troublesome symptoms.

5.00

I have no symptoms — **extremely troublesome or severe symptoms**

How frequently did you use antihistamines and/or corticosteroids in tablets and/or nasal sprays during the **year before** the start of treatment? Mark a point on the line below, bearing in mind that the left end indicates that you never used medicinal products and the right end indicates that you used them frequently.

5.00

never used — **frequently used**

Before the start of treatment how long did your symptoms last?

- ☐ more than 4 days a week
- ☐ more than 4 weeks

Before the start of treatment, did the allergy disturb your sleep?

- ☐ Yes
- ☐ No

Before the start of treatment, did the symptoms limit your normal work or school activities and affect your performance?

- ☐ Yes
- ☐ No

Before the start of treatment, were your symptoms little troublesome ?

- ☐ Yes
- ☐ No

Before the start of treatment, were your symptoms severe?

- ☐ Yes
- ☐ No

CONTINUED

WE SHALL NOW ASK YOU SOME QUESTIONS ABOUT THE FIRST YEAR OF TREATMENT WITH SPECIFIC SUBCUTANEOUS IMMUNOTHERAPY

RHINOCONJUNCTIVITIS

What was the severity of nose and eye symptoms during the **first year** of treatment? Mark a point on the line below, bearing in mind that the left end indicates that you had no symptoms and the right end indicates that you suffered extremely troublesome symptoms.

5.00

I have no symptoms — **extremely troublesome or severe symptoms**

How frequently did you use antihistamines and/or corticosteroids in tablets and/or nasal sprays during the**first year** of treatment? Mark a point on the line below, bearing in mind that the left end indicates that you never used medicinal products and the right end indicates that you used them frequently.

5.00

never used — **frequently used**

CONTINUED

WE SHALL NOW ASK YOU SOME QUESTIONS ABOUT THE END OF TREATMENT WITH SPECIFIC SUBCUTANEOUS IMMUNOTHERAPY

RHINOCONJUNCTIVITIS

What was the severity of nose and eye symptoms at the **end** of the treatment? Mark a point on the line below, bearing in mind that the left end indicates that you had no symptoms and the right end indicates that you suffered extremely troublesome symptoms.

5.00

I have no symptoms **extremely troublesome or severe symptoms**

How frequently did you use antihistamines and/or corticosteroids in tablets and/or nasal sprays at the **end** of treatment? Mark a point on the line below, bearing in mind that the left end indicates that you never used medicinal products and the right end indicates that you used them frequently.

5.00

never used **frequently used**

CONTINUED

WE SHALL NOW ASK YOU QUESTIONS ABOUT THE PERIOD OF DISCONTINUATION OF THE TREATMENT

How many years ago did you discontinue treatment?

- ☐ 1 year
- ☐ 2 years

RHINOCONJUNCTIVITIS

What was the severity of nose and eye symptoms **since you discontinued** the treatment? Mark a point on the line below, bearing in mind that the left end indicates that you had no symptoms and the right end indicates that you suffered extremely troublesome symptoms.

5.00

I have no symptoms — **extremely troublesome or severe symptoms**

How frequently did you use antihistamines and/or corticosteroids in tablets and/or nasal sprays been **since you discontinued** the treatment? Mark a point on the line below, bearing in mind that the left end indicates that you never used medicinal products and the right end indicates that you used them frequently.

5.00

never used — **frequently used**

How long have your symptoms lasted **since you discontinued** the treatment?

- ☐ more than 4 days a week
- ☐ more than 4 weeks
- ☐ never

Have you slept well **since you discontinued** the treatment?

- ☐ Yes
- ☐ No

Have the symptoms limited your normal work or school activities and affected your performance **since you discontinued** the treatment?

- ☐ Yes
- ☐ No

Have your symptoms been little troublesome **since you discontinued** the treatment?

- ☐ Yes
- ☐ No

Have your symptoms beensevere **since you discontinued** the treatment?

- ☐ Yes
- ☐ No

CONCLUSIVE QUESTIONS

Are you satisfied with the treatment?

- ☐ Very unsatisfied
- ☐ Unsatisfied
- ☐ Satisfied
- ☐ Very satisfied

Indicate with a score from 1 to 5 you badly you have been disturbed during the past 2 weeks by:

	Not at all	A little	Quite a lot	A lot	A great deal
Blocking, dripping or itchy nose	1	2	3	4	5
Itching, lacrimation, burning or redness of the eyes	1	2	3	4	5
Difficulty concentrating	1	2	3	4	5
Wheezing, coughing, chest tightness or difficulty breathing	1	2	3	4	5
Disturbed sleep (e.g. night waking)	1	2	3	4	5
Having to avoid certain environments	1	2	3	4	5
Having to take medicinal products	1	2	3	4	5
Limitations to normal daytime activities (work, study, sport)	1	2	3	4	5

Enter your check code to complete the questionnaire

COMPLETE QUESTIONNAIRE
