# Supplementary material for: Real-Life Effectiveness of Subcutaneous Immune Therapy with Carbamylated Monomeric Allergoids on Mite, Grass, and Pellitory Respiratory Allergy: A Retrospective Study
Source: J Clin Med. 2022 Dec 12;11(24):7384. doi: 10.3390/jcm11247384 (PMC9781390; doi:10.3390/jcm11247384)
Supplement: Supplementary file 1 [file jcm-11-07384-s001.zip › supplementary files/supplementary file 1 _data shit.pdf]

SUMMARY OF PRODUCT CHARACTERISTICS

1. NAME OF THE MEDICINAL PRODUCT

Lais-*in* 10 BU/mL**.** Suspension for injection.
Chemically modified allergenic extract (momoneric allergoid)

2. QUALITATIVE AND QUANTITATIVE COMPOSITION

Active substances: chemically modified extract (carbamylated monomeric allergoid) of house-dust mites (*Dermatophagoides pteronyssinus* 50% and *D. farinae* 50%), Grass pollen (*Holcus lanatus* 33%, *Phleum pratense* 33%, *Poa pratensis* 33%) and Parietaria pollen (*Parietaria officinalis* 50%, *Parietaria judaica* 50%) in a suspension for subcutaneous injections adsorbed on calcium phosphate, as per individual prescription. Concentration: 10 BU/mL equivalent to:

- 4 µg* Equivalent/ml of major allergens Group 1 (Der p 1 and Der f 1) of house-dust mites;
- 5 µg Equivalent/ml of major allergens Group 5 (Phl p5, Hol l5 and Poa p5) of Grass pollen;
- 1,5 µg Equivalent/ml of major allergens Group 1 (Par j 1 and Par o 1) of Parietaria pollen.

* µg determined on the purified extract before chemical modification

The biological unit of the subcutaneous specific immunotherapy Lais-*in* is the Biological Unit **BU/mL**, standardised on the basis of cutaneous tests in sensitised subjects. The allergenic potency of each production batch is kept consistent by means of the EAST-inhibition technique in comparison to an In House Reference Preparation-IHRP.

The BU is a drug unit equivalent to 1/100 of the concentration of extract which, before being chemically modified, induces a mean wheal equivalent to that induced by 10 mg/mL histamine during skin allergy testing.

For a full list of excipients see section 6.1.

3. PHARMACEUTICAL FORM

Suspension for injection.

4. CLINICAL PARTICULARS

4.1. THERAPEUTIC INDICATIONS

Treatment of type I IgE-mediated allergic diseases such as rhinitis, conjunctivitis and bronchial asthma.

4.2. POSOLOGY AND METHOD OF ADMINISTRATION

The specific immunotherapy with Lais-*in* is administered by a physician by injection. The injection is subcutaneous at the back or side of the upper arm, after carefully disinfecting the skin. Prior to drawing up the quantity of allergen to be injected, softly shake the vial until forming a homogenous, foam-free suspension.

The dosage regimen is individualised based on the clinical progress and tolerability of the patient. Therefore the treatment schedule suggested has been provided for guidance only and may be adapted to the patient's needs by the physician administering the therapy.

Induction phase

Start the treatment with the lowest dose and gradually increase the doses only when the last administered dose was well-tolerated. The interval between administrations is 1 week during the induction phase.
A usual dosage regimen during the **induction phase** (the vial contains 10 BU/mL) may be:

Concentration	Treatment week	Dose in mL
10 BU/mL	1	0.10
	2	0.20
	3	0.30
	4	0.50

The dosage regimen allows the patient to reach the maximum tolerated dose, which may correspond to the maximum dose of 0.70 mL.

Maintenance Treatment

Once the maximum tolerated dose has been reached, repeat the treatment every 4 weeks unless otherwise prescribed by the physician.
In case of seasonal allergens such as pollens, it is recommended to start the treatment at least 2 months before pollen season and continue it following the physician instructions.
In case of perennial allergens such as house-dust mites, treatment may start at any time of the year and may be continued without interruption after the maximum tolerated dose has been reached.
It is recommended that the treatment continues for a period of 3-5 consecutive years.

4.3. CONTRAINDICATIONS

- Chronic or acute inflammatory fever diseases
- Active tuberculosis
- Pathologic shock organ affections (e.g. emphysema, bronchiectasis)
- Immunodeficiencies (e.g. also from immunosuppressive therapy)
- Autoimmune diseases
- Malignant tumors
- Serious and uncontrolled bronchial asthma, in particular with a FEV_1 value persistently lower than 70%
- Cardio-vascular diseases, treatment with beta-blockers
- Conditions in which the use of adrenaline is contraindicated (e.g. hyperthyroidism)
- Insufficient compliance to the therapy by the patient
- Hypersensitivity to one of the excipients (see section 6.1).

4.4. SPECIAL WARNINGS AND PRECAUTIONS FOR USE

Subcutaneous immunotherapy should only be administered by a physician who has immediate access to appropriate emergency room.
After the injection, the patient should undergo medical surveillance for at least 30 minutes.
The physician should carefully check the dose to be administered to the patient before extracting the amount of allergen to be injected from the vial.
Do not administer the drug intradermally, intravenously or intramuscularly.
The subcutaneous injection should be performed slowly by using a graduated 1 mL syringe. Introduce the needle under the skin and pull the plunger back on the syringe before injecting the allergen to make sure that the needle has not accidentally penetrated a vein; for higher doses, it is recommended to aspirate the air by pulling the plunger back once again while injecting. If the needle has accidentally penetrated a vein, immediately withdraw the syringe and wait 20-30 minutes before using a new syringe to inject into another site. Do not massage the injection site after the injection.
It is recommended to write down the injected dose, date, the injection site and any undesirable effects in a diary.
If treatment is interrupted for a week during the induction phase, repeat the last dose tolerated by the patient. If treatment is interrupted for two or more weeks, reduce the injected dose by 50% and increase the dosage weekly until the maximum recommended or tolerated dose is reached. Use the same precautions during maintenance therapy each time a new vial is used.

If the patient needs any type of anti-pathogenic, antiviral or anti-bacterial vaccination, wait at least 4-5 days between the last administration of the immunotherapy and the vaccination. The immunotherapy may continue approximately for 2 weeks after vaccination. A physician should be consulted in case infections and/or inflammations occur.
Alcohol, intense physical activity, hot baths and saunas should be avoided immediately after the injection.

4.5. INTERACTIONS WITH OTHER DRUGS

There are no known interactions with other drugs.

4.6. PREGNANCY AND BREASTFEEDING

Pregnancy

Specific immunotherapy should not be used during pregnancy, mainly because the use of drugs is restricted at that time. If interrupting the specific immunotherapy poses a risk for the patient, the physician should decide whether to continue after carefully evaluating the risks incurred by the patient from being unprotected.

Breastfeeding

There are no contraindications as for the treatment during breastfeeding.

4.7. EFFECTS ON THE ABILITY TO DRIVE AND USE MACHINES

Similar to other types of immunotherapy, the patient may experience fatigue after administration.
This could lower the threshold of attention in patients, thus affecting their ability to drive and/or using machines.

4.8. UNDESIRABLE EFFECTS

Undesirable effects may occur, generally at organ level, such as rhinitis and watering eyes, or locally at the injection site, such as swelling and itching. Delayed reactions several hours after administration of the vaccine may occur. Systemic anaphylactic reactions are rare. The occurrence of local and/or systemic side effects during specific immunotherapy should be carefully evaluated by the physician who will adjust the dosage and, if necessary, or in case of anaphylactic reaction, prescribe suitable anti-allergy treatment depending upon the severity of the clinical situation (oral and/or parenteral anti-histamines; oral and/or parenteral corticosteroids; beta-2 adrenergic agonists; subcutaneous adrenaline).

4.9. OVERDOSE

An overdose may cause local or systemic allergic reactions. Symptoms should be controlled by anti-allergy drugs (anti-histamines; corticosteroids; subcutaneous adrenaline) based on the severity of the situation and the opinion of the physician.

5. PHARMACOLOGICAL PROPERTIES

5.1. PHARMACODYNAMIC PROPERTIES

Pharmacotherapeutic category: Allergens. Code ATC V01A
Specific Immunotherapy is a therapeutic mode characterized by the repeated administration of specific allergens with the aim of modifying the immunological response of the patient suffering from conditions definitely related to an allergic pathogenesis, as it significantly interferes with IgE-mediated inflammatory reactions that occur after exposure to natural allergens. Such therapeutic mode, consolidated in allergy clinics, has the aim of protecting the patient suffering from pathogenetic allergy-related diseases to the specific allergen and eventually modifying the natural course of the disease. Among the action mechanisms explaining the clinical efficacy there are the increasing of the so-called IgG4 subclass antibodies, a pathogenetically significant reduction of the seasonal peak of specific IgEs, a reduction of allergen-specific lymphocyte proliferation.
In the last years solid experimental evidence has been provided, which indicate that this clinical practice is capable of rebalancing the T- helper lymphocyte cytokine profile of CD4 phenotype causing a functional “switch” from the prevailing phenotype Th2 to the phenotype Th1.

The adsorption on calcium phosphate decreases the presence of free allergen, allows a constant allergenic effectiveness and a gradual and constant release of the allergen itself.
Lais–*in* contains chemically modified allergens (**allergoids**) of house-dust mites *(Dermatophagoides pteronyssinus* 50% and *Dermatophagoides farinae* 50%) or chemically modified extract of Grass pollen (*Holcus lanatus* 33%, *Phleum pratense* 33%, *Poa pratensis* 33%) or chemically modified extract of Parietaria pollen (*Parietaria officinalis* 50%, *Parietaria judaica* 50%). This chemical modification, obtained by the carbamylation at alkaline pH, determines a significant reduction of the capability to react with IgE antibodies, and, subsequently, reduces the allergenicity of the product, allowing a safer administration.

6. PHARMACEUTICAL INFORMATION

6.1. LIST OF EXCIPIENTS

Sodium chloride 7.8 mg/ml, phenol 4 mg/ml, calcium phosphate up to 1.3 mg/ml, water for injectable solutions as required for 1 mL.

6.2. INCOMPATIBILITIES

No incompatibilities with other drugs are known.

6.3. SHELF LIFE

18 months. The expiration date printed on the package applies to the product properly stored in the original package.

6.4. SPECIAL PRECAUTIONS FOR STORAGE

Store in a refrigerator at a temperature between 2°C and 8°C from reception. Do not freeze the product. Once frozen, the product cannot be used anymore.

6.5. NATURE AND CONTENT OF THE CONTAINER

A package containing 1 or 2 vials with 3.5 mL of chemically modified extract at a concentration of 10 BU/mL is equivalent to:
4 µg Equivalent/ml of major allergens Group 1 (Der p 1 and Der f 1) of house-dust mites;
5µg Equivalent/ml of major allergens Group 5 (Phl p5, Hol l5 and Poa p5) of Grass pollen;
1,5 µg Equivalent/ml of major allergens Group 1 (Par j 1 and Par o 1) of Parietaria pollen.

6.6. SPECIAL PRECAUTIONS FOR DISPOSAL

Any unused medicinal product or waste material should be disposed of in accordance with local requirements.

7. MARKETING AUTHORISATION HOLDER

LOFARMA S.p.A.
Viale Cassala 40
20143 Milano
ITALIA

8. MARKETING AUTHORISATION NUMBER

-

9. DATE OF FIRST AUTHORISATION

The marketing authorisation is in accordance with: Italian Legislative Decree 13.12.1991 “Disposizioni sui radiofarmaci e sugli allergeni”.

10. DATE OF REVISION OF THE TEXT

Aprile 2014
